# A Systematic Review and Meta-Analysis of Health Utility Estimates in Chronic Spontaneous Urticaria

**DOI:** 10.3389/fmed.2020.543290

**Published:** 2020-12-09

**Authors:** Yan Yuan, Yi Xiao, Xiang Chen, Jie Li, Minxue Shen

**Affiliations:** ^1^Department of Dermatology, Xiangya Hospital, Central South University, Changsha, China; ^2^Hunan Engineering Research Center of Skin Health and Disease, Central South University, Changsha, China; ^3^Hunan Key Laboratory of Skin Cancer and Psoriasis, Central South University, Changsha, China; ^4^Department of Social Medicine and Health Management, Xiangya School of Public Health, Central South University, Changsha, China

**Keywords:** chronic spontaneous urticarial, utility-estimate, disease burden, quality of life, health-related burden

## Abstract

**Background:** Chronic spontaneous urticaria (CSU) is a common recurrent skin disease that adversely affect patient's quality of life condition to treat. Economic evaluations of health care often include patient preferences for health outcomes using utilities.

**Objectives:** The study aimed to determine pooled estimates of utility-based quality of life in patients with CSU.

**Methods:** We conducted a systematic review, meta-analysis, and meta-regression of peer-reviewed articles and conference papers that published from database inception to 31 April 2019 that reported utility estimates in patients with CSU. Scores reported with the EQ-5D, SF-6D, SF-12, and SF-36 instruments were converted to utilities using published mapping algorithms. Meta-analysis was used to calculate the pooled and meta-regression was used to examine the effects of possible factors.

**Results:** The pooled utility estimate for CSU was 0.68 [95% confidence interval (CI): 0.67–0.70]. The pooled utility estimate that converted from SF-36 or SF-12 was 0.66 (95% CI: 0.58–0.74), 0.72 (95% CI: 0.70–0.74) for EQ-5D, and 0.65 (95% CI: 0.63–0.67) for SF-6D, respectively. According to the meta-regression, higher proportion of female patients was significantly associated lower utility estimates (*p* = 0.013).

**Conclusions:** The study provides evidence-based utility estimates to inform health-related burden analysis of CSU and reference for the follow-up cost-effectiveness evaluation of chronic spontaneous urticaria intervention. These results highlight differences in common utility-based instruments and need to be cognizant of the specific instruments used when comparing the results of outcome studies.

## Introduction

Chronic urticaria (CU) is defined as the spontaneous appearance of wheals, angioedema, or both, for more than 6 weeks due to known and unknown causes. CU can be divided into chronic spontaneous urticaria (CSU) and chronic inducible urticaria (CIU) (1). There are no identifiable triggers for CSU, and symptoms and signs can be unpredictable. Recent studies have reported that the point prevalence of CU diagnosis in the United States and five European countries is 0.53 and 0.63%, respectively (2–4). The disease is most common in patients aged 20–40 years, but can be observed in all age groups (5). Studies have consistently shown that women are almost twice as likely as men to develop the disease (3, 4, 6, 7).

CSU has an enormous impact on an individual's quality of life, which is attributable in part to swelling, itching, pain, and fatigue. Sleep disorders can also lead to poor health status in CSU, which is often caused by itching that comes with hives (8–11). Mental health can also be affected because the unpredictability of outbreaks can create a sense of loss of control (12). Patients also reported emotional and social problems, such as depression, anxiety, family management, personal care, recreational or social activities, and mobility.

Quality of life estimates (utilities) is essential for economic evaluation because the quality of life is a critical component of economic benefits. Quality-adjusted life years are a measure of a person's length of life, which is an assessment of their health-related quality of life during that time. Quality-adjusted life years are the preferred outcome of cost-effectiveness studies. Preference-based instruments derived from utility score, anchored at 1 for perfect health and 0 for death (13). Although negative numbers are possible, and they reflect health states considered worse than death. The utility can be measured by several approaches, using direct methods such as time trade-off and standard gamble, and multi-attribute utility instrument such as the EuroQoL group's EQ-5D, the UK's SF-6D, and the 15D from Finland. Besides, data from generic non-utility-based quality of life instruments such as Short Form-36 (SF-36) and the Short Form-12 (SF-12) can be converted to a utility estimate using published transformation algorithms (14, 15). The EQ-5D evaluates the health status through 243 distinct health states across five dimensions (mobility, self-care, usual activities, pain/discomfort, and anxiety/depression), from which HSUVs can be derived based on different population norms.

There are various tools to measure utility-based quality of life. In recent years, meta-analysis has recommended as a strategy to generate overall utility values for common health states, which has included studies of utility values for HIV/AIDS (16), chronic kidney disease (17), diabetes (18), osteoporosis (19), and cancers (20, 21). The results of the meta-analysis were more concentrated than those of descriptive analysis. The purpose of this study is to systematically evaluate the methods and results of the health utility value of CSU-related disease states, and to provide data reference for subsequent utility evaluation.

## Methods

### Study Selection

Our systematic review was prospectively registered on PROSPERO (http://www.crd.york.ac.uk/PROSPERO; registration number CRD42020147713) and followed the PRISMA checklist for reporting (Supplementary Table 1). We searched PubMed, Embase, Web of Science, the Cochrane Database of Systematic Reviews, the MeSH terms, and text words used are provided in Supplementary Table 2.

Studies were included if their study participants had CSU and aged above 18 years. Conference papers were also included if sufficient data for analysis were provided. We included all studies that either reported utility directly or estimated utility from SF-36 or SF-12 health surveys using peer-reviewed algorithms. Studies that reported estimates from health-related quality of life scores were also excluded unless all eight SF-36 domains were reported separately, and a utility estimate could be calculated, as above.

### Data Extraction and Management

Data from included studies were extracted independently by two reviewers, with differences resolved through discussion. Data on the principal author, year of publication, clinical and demographic characteristics of patients, number and country of patients, study design, method used to report a utility value and its estimate (mean and SD) were captured.

### Study Quality Assessment

Quality was assessed using the Newcastle-Ottawa scale (NOS) (22) for inclusion in case-control studies and cross-sectional studies. The NOS includes a set of items for assess the selection of study participant, the comparability of population, and the determining exposure or outcome with a maximum score of 9. Studies with a score of 5 or more were classified as high-quality studies (23). Using these checklists, two reviewers assessed the quality of each article included. Differences of opinion were resolved through consultation with a third reviewer.

### Data Analysis

The command “metan” was used to conduct meta-analysis using the STATA version 14. Heterogeneity among the studies was evaluated using the *I*^2^ statistic. Fixed effect model was used if *I*^2^ < 50%; otherwise the random effect model was used. Pooled utility estimates and 95% confidence intervals (CIs) were calculated and displayed as forest plots. We performed subgroup analyses by utility elicitation approach. Significance of subgroups was determined by Wald test (24). Meta regression was performed to examine the effect of gender on utility estimates. Publication bias may arise in this field of investigation, so we tested publication bias using the Begg and Egger test.

## Results

### Study Selection

**Figure 1:**
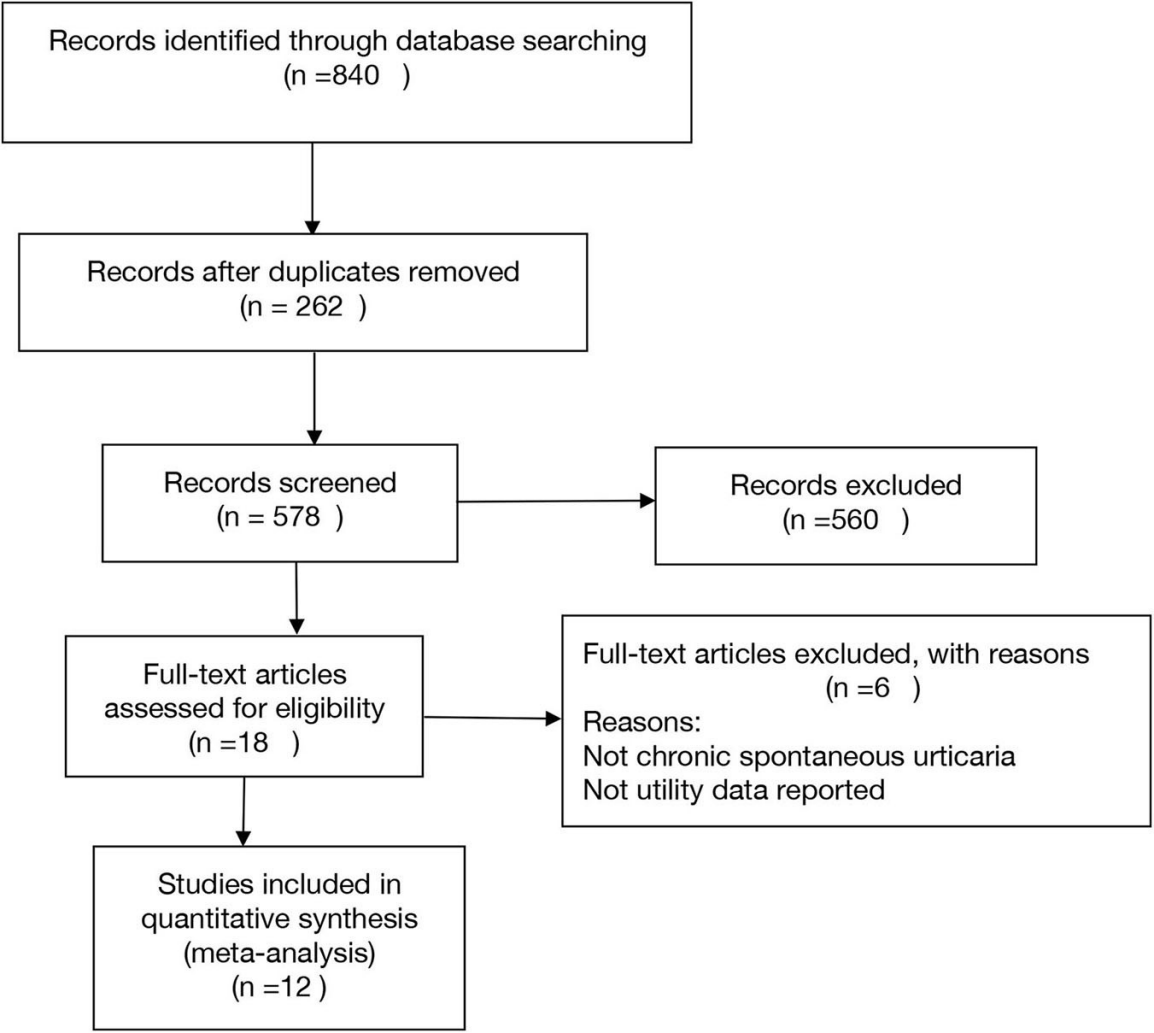
Flow diagram for study selection.

Initially, the electronic database searches identified 578 tittles after duplicates were removed. There were 49 articles and abstracts remaining after title screening. Finally, after screening by abstract and full text, 12 articles remained (Figure 1). It was representing 4,155 patients (Table 1). A total of 17 utility estimates were identified from the included studies (Table 2).

**Table 1:** Characteristics of the included studies.

**References**	**Country**	**Study design**	**Participants**	**Female (%)**	**Age**		**Utility elicitation method**	**Quality assessment**
					**Mean**	**SD**		
Wilson (25)	Italy	Case-control	21	76.19	46.30	12.40	EQ-5D derived from SF-36	7
Baiardini et al. (26)	Turkey	Case-control	84	85.00	36.83	10.26	EQ-5D derived from SF-36	8
Ozkan et al. (27)	EU	Case-control	343	N/A	N/A	N/A	SF-6D	6
Balp et al. (28)	EU	Cross-section	369	65.00	44.40	15.20	SF-6D	7
Balp et al. (29)	Canada	Cross-section	88	N/A	N/A	N/A	EQ-5D	3
Balp et al. (29)	Germany	Cross-section	98	N/A	N/A	N/A	EQ-5D	3
Balp et al. (29)	UK	Cross-section	79	N/A	N/A	N/A	EQ-5D	3
Balp et al. (29)	Netherlands	Cross-section	99	N/A	N/A	N/A	EQ-5D	3
McBride et al. (30)	Canada	Cross-section	86	N/A	N/A	N/A	EQ-5D	3
Sussman et al. (31)	US	Case-control	270	74.00	48.50	15.20	SF-6D	8
Vietri et al. (32)	UK	Randomized trials	318	13.00	41.20	14.50	EQ-5D	5
Vietri et al. (32)	UK	Randomized trials	322	22.00	42.50	13.70	EQ-5D	5
Vietri et al. (32)	UK	Randomized trials	335	6.00	43.10	14.10	EQ-5D	5
Hawe et al. (33)	Brazilian	Cross-section	127	65.00	37.10	11.80	SF-6D	7
Balp et al. (34)	US	Cross-section	747	74.00	50.50	15.70	SF-6D	6
Mendelson et al. (35)	5EU	Cross-section	769	71.00	45.40	15.20	SF-6D	6
Sau Huu et al. (36)	Vietnam	Cross-section	44	10.20	N/A	N/A	EQ-5D	7

**Table 2:** Utility values estimated in included studies.

**References**	**Utility elicitation method**	**Utility value Mean (SD)**	
		**Mean**	***SD***
Wilson (25)	EQ-5D from SF-36	0.72	0.27
Baiardini et al. (26)	EQ-5D from SF-36	0.63	0.25
Ozkan et al. (27)^*^	SF-6D	0.63	0.20
Balp et al. (28)^*^	SF-6D	0.63	0.20
Balp et al. (29) Canada	EQ-5D	0.71	0.30
Balp et al. (29) Germany	EQ-5D	0.71	0.25
Balp et al. (29) UK	EQ-5D	0.72	0.31
Balp et al. (29) Netherlands	EQ-5D	0.76	0.27
McBride et al. (30)	EQ-5D	0.70	0.30
Sussman et al. (31)^*^	SF-6D	0.64	0.20
Vietri et al. (32) ASTERIA I	EQ-5D	0.68	0.27
Vietri et al. (32) ASTERIA II	EQ-5D	0.71	0.26
Vietri et al. (32) GLACIAL	EQ-5D	0.73	0.24
Hawe et al. (33)^*^	SF-6D	0.64	0.26
Balp et al. (34)	SF-6D	0.67	0.15
Mendelson et al. (35)^*^	SF-6D	0.67	0.18
Sau Huu et al. (36)^*^	EQ-5D	0.78	0.17

* *Date of SD were imputed by the standard deviation = 0.368 – 0.82 × UtilityScore^2^ + 0.625 × UtilityScore^3^*.

### Study Characteristics

Studies were conducted in a variety of countries. Not all of the studies reported in the 11 publications had large sample sizes (ranged from 21 to 769 participants). The average age of participants across all studies varied between 36 and 51 years. In all but three studies, more than half of the participants in each study were women (Table 1).

### Quality Assessment

Although one study was a randomized trial, we used the baseline data only and hence treated it as an observational study in the quality assessment. The bias risk assessment results of observational studies show that the overall quality of the 9 studies is good, and about 7/9 items are reflected. The main problems are the Ascertainment of exposure, as shown in Supplementary Table 3.

### Imputation of Standard Deviations

The standard deviation was available for 13 (76.5%) utility estimates. When the standard deviation of a utility estimate was not reported, it was calculated from the standard error. For utilities derived from the SF-12 and SF-36 instruments, we used the method which fitted a regression model of the observed standard deviations against utility estimates (17), and then used the estimated equation to predict standard deviations when they were not available.

### Health State Utility Value Outcomes

**Figure 2:**
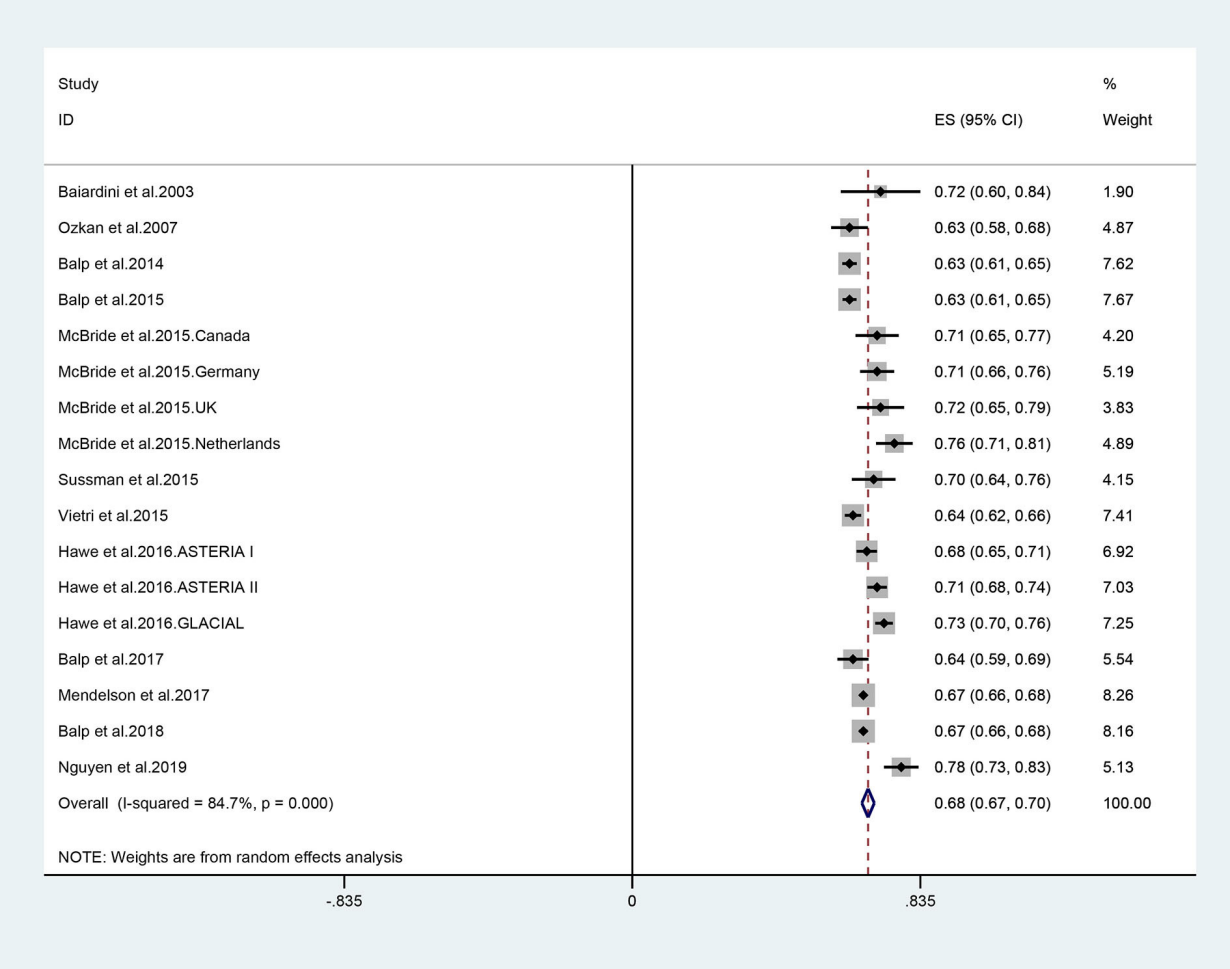
Forest plot (random effect) of utility estimates elicited of chronic spontaneous urticaria.

**Figure 3:**
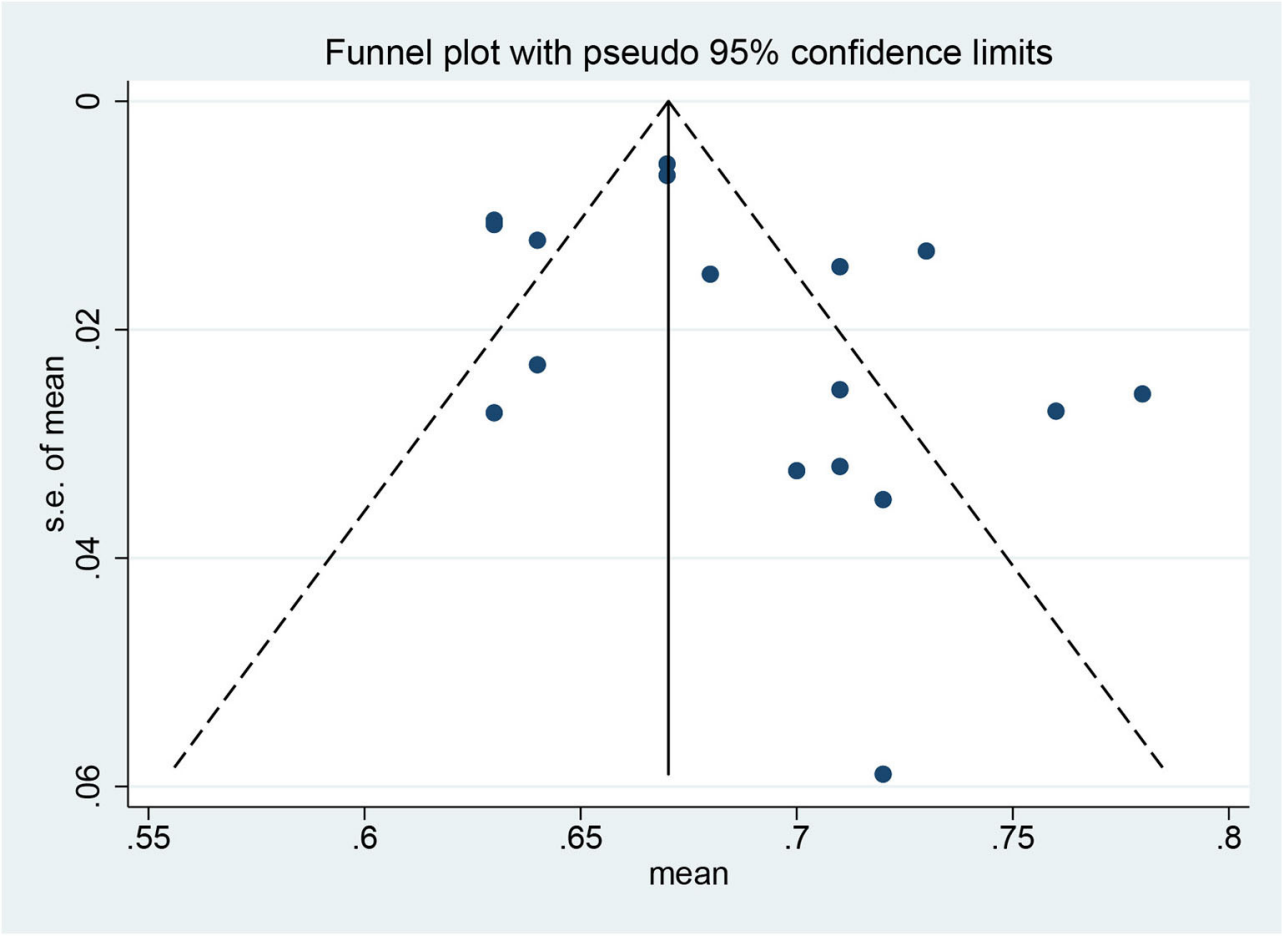
Funnel plot of meta-analysis for adjusted studies.

The utility for CSU was estimated to be 0.68 (95% CI: 0.67–0.70) based on the random effect model, and the *I*^2^ statistic was 84.7% (Figure 2). No significant changes in pooled estimates were identified in the sensitivity analysis by excluding one study at a time (Supplementary Figure 1). According to the funnel plot (Figure 3) and the results of Begg test (*P* = 0.202) and egger test (*P* = 0.187), there was no significant publication bias in these studies. The difference in utility values between men and women at CSU was reported in only one study (36), which is a cross-sectional study on quality of life in different skin diseases in Vietnam. The utility value of EQ-5D for CSU was 0.76 in male, 0.80 in female, and 0.78 overall. No other study reported on the utility value by gender.

### Subgroup Analysis

**Figure 4:**
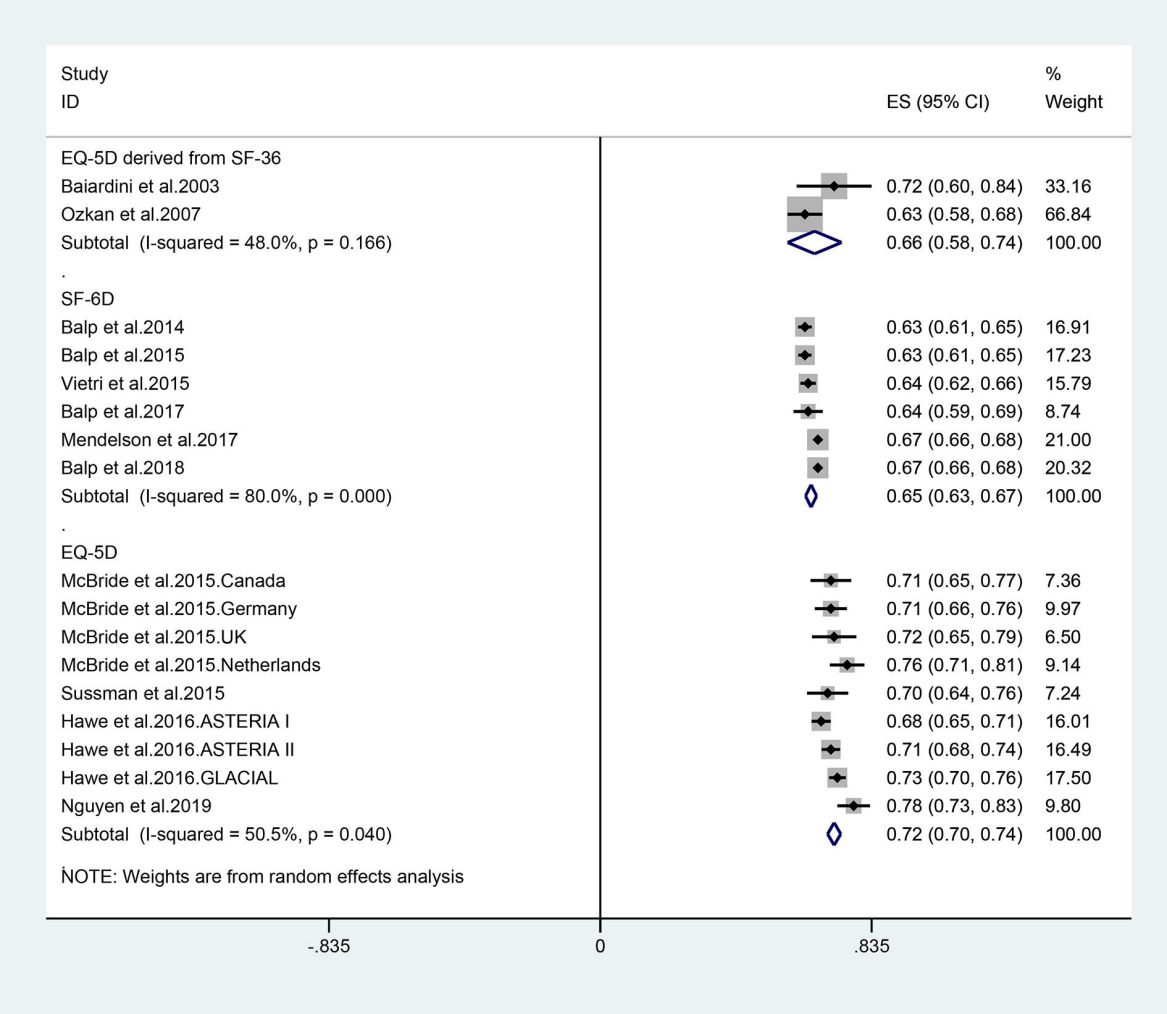
Forest plots of utility estimates for patients with different utility elicitation approach.

We performed the subgroup analysis by utility elicitation instrument. There were slight differences in utility between the EQ-5D deprived from SF-12/36, and EQ-5D directly. The utility scores were similar between the three instruments: the pooled utility estimate was 0.66 (95% CI 0.58–0.74) converted from the SF-36 or SF-12 questionnaires, 0.72 (95% CI: 0.70–0.74) mapped from the EQ-5D, and 0.65 (95% CI: 0.63–0.67) mapped from the SF-6D (Figure 4).

### Meta Regression

**Figure 5:**
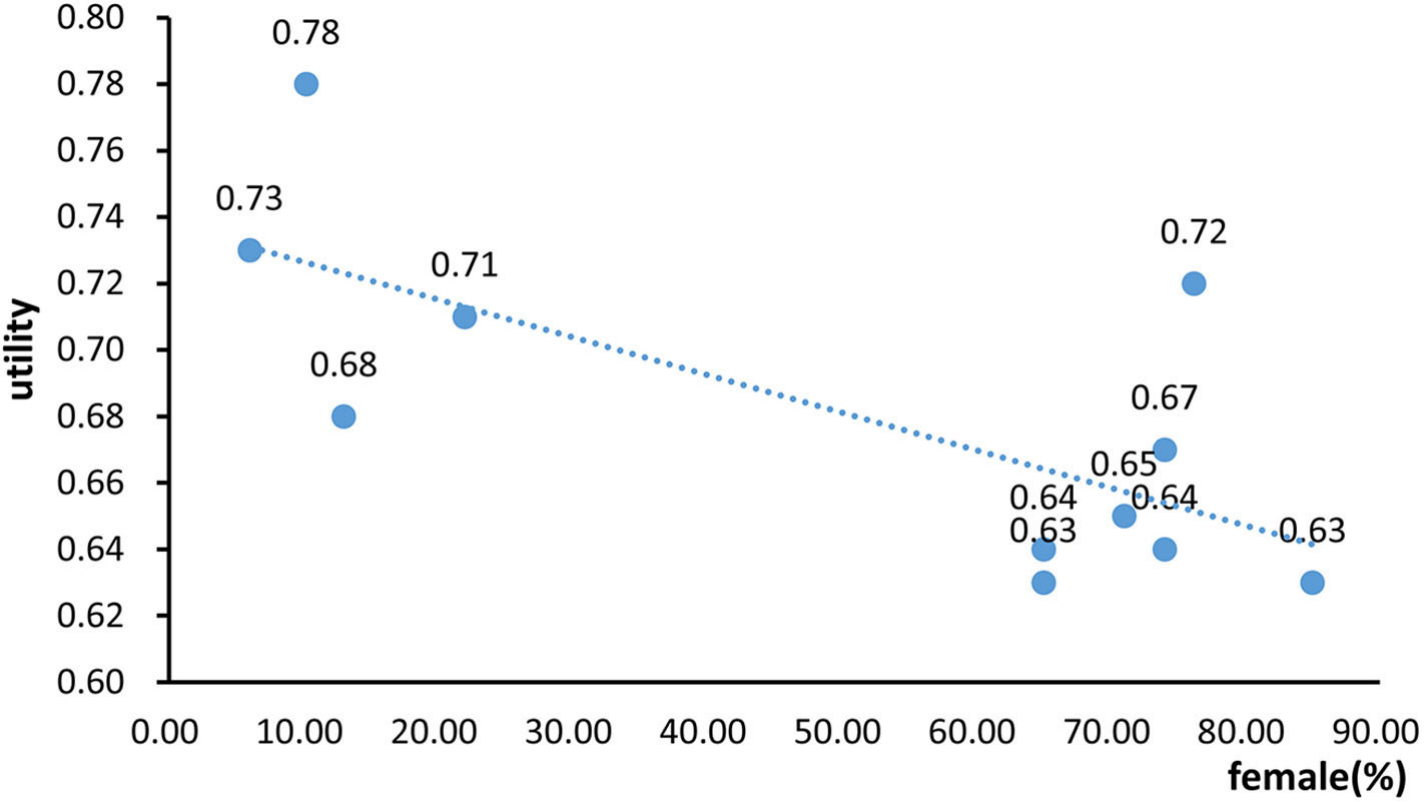
The regression model of the association between proportion of female patients with chronic spontaneous urticaria utilities.

Meta regression showed that the proportion of female patients was significantly inversely correlated with the CSU utilities (*R*^2^ = 0.5108, *p* = 0.013). Higher proportion of female patients was significantly associated lower utility estimates (Figure 5).

## Discussion

Our meta-analysis showed that the pooled estimate of utility for CSU was 0.68. The utility estimate mapped from EQ-5D was slightly higher than that derived from other tools and mapped from SF-6D. The Meta-regression showed that female was associated with lower utility estimates.

The systematic review collected the values that were reported in the literature, providing a synthesis of appropriately reported values through the meta-analysis. The study has several strengths. First, to our knowledge, this study is the first to estimate a pooled utility value from studies in patients with CSU. The second strength of this review is its size and comprehensiveness, consisting of 12 studies that including 17 utility estimates from over 4,100 participants from more than 10 countries. Third, we included different utility elicitation instruments, enabling comparisons across the instruments. Finally, in this review, the applicability of the utility estimates obtained from the EQ-5D and SF-6D was discussed when evaluating the difference in the quality of life of CSU. Published studies focused more on discussing how to use SF-6D better to predict EQ-5D scores for economic evaluation, and rarely discussed the applicability of these two instruments.

There are limitations applied to this research. First, our meta-regression model failed to investigate the effect of other potential confounders such as disease severity, age, education, comorbidity, socioeconomic status, or race/ethnicity on utility values, due to the lack of information from qualified original articles. Second, we calculated EQ-5D scores from SF-36 and SF-12 data, and the algorithms may not reflect what the EQ-5D scores measured directly. This is of particular note given a large number of studies that used SF-36 and SF-12. Additionally, some of the standard deviations were imputed, and this might affect the precision of the pooled estimate. Therefore, the results should be considered as suggestive rather than definitive. The performance of the selected algorithm and limitations need to be further evaluated in future studies.

According to our study, the mean utility estimate from SF-6D is lower than that from EQ-5D. There are several possible explanations. First, compared with the EQ-5D, the SF-6D has an additional dimension of vitality. Fatigue lack of sleep or insomnia are commonly reported in patients with CSU due to its constant itch accompanied by the collapse of urticarial (37–39), which has considerable influence on the dynamic of vitality. Second, the two instruments have different foci. EQ-5D focuses on the classification of the severity of health conditions, and each dimension is defined as three levels (no problems, some problems, and extreme problems). In contrast, SF-6D is more concerned about the duration of adverse effects in health status or the degree of impact on life, and each dimension has four to six levels, such as “all the time” and “most of the time.” SF-36 or SF-12 questionnaires should be used to collect data when SF-6D applied. Although CSU is not life-threatening, it has been demonstrated to have a substantial impact on the physical and psychological health of patients with long term courses (28, 31, 40). Third, EQ-5D measures the health status of respondents on the day they complete the questionnaire; however, items of SF-36 or SF-12 covered a survey period of “the past 4 weeks,” participants were asked to think about their health in the past; this may result in a more comprehensive evaluation in general. Fourth, a serious ceiling effect of EQ-5D has been reported previously. Vietri et al. (41) investigated the quality of life of 346 respondents using EQ-5D and SF-6D. The results showed that 107 patients were in full health condition as determined by EQ-5D. While only 17% of the patients reported no “pain or discomfort,” 89% reported no problem of “self-care ability.” Similarly, the floor effect of SF-6D is prominent. For example, only 24.6 and 38.4% reported unaffected “body function” and “role limitation,” respectively. In short, EQ-5D is less sensitive to indicate relatively good health status, while SF-6D is less discriminative to indicate poor health status. Sensitivity often is a criticism of the EQ-5D in terms of its limited ability to distinguish between subtle differences or changes of the disease.

With respect to the characteristics of CSU including long course, easy recurrence, and a significant influence on mental condition, SF-12/36 questionnaires might be a better choice to understand the changes in the quality of life in CSU patients in the long process from the initial onset to a gradual recovery. The selection of suitable HRQOL instruments remains a challenge, depending on the objectives of study, psychometric characteristics of the instrument, and the study population.

The meta-regression showed that a higher proportion of female patients was associated with lower utility estimates. This indicated that women might report higher levels of anxiety and depression disorders than men surrounding skin disorders. Disfigurement is a more sensitive issue for women than men, and skin disorders could develop more disfigurement-related psychological distress for women (37, 42–44). A study suggested that the gender differences in genetic, metabolic, sociodemographic, socioeconomic status, and biomarkers such as histamine might also contribute to the higher risks of developing depression and anxiety disorders in females (45). In addition, females have lower pain thresholds and tolerance, more pain-seeking treatment, which may explain the lower utility estimates compared to males (46, 47).

Several recommendations can be drawn from the findings of this study. First, difference means among different measuring tools suggest that we should wisely use the tools according to both characteristics of the tool and the targeted diseases. Second, gender disparities in utility estimates of CSU suggest that more attention should be paid to female patients. Most importantly, our study provides the mean utilities, which can be applied in future health economic evaluations investigating the disease burden of CSU.

## Data Availability Statement

The original contributions presented in the study are included in the article/Supplementary Material, further inquiries can be directed to the corresponding author/s.

## Author Contributions

YY and YX contributed to the conception and design the article, and wrote the manuscript. YY, YX, MS, and JL analyzed the data. JL, XC, and MS reviewed the manuscript. All authors contributed to the article and approved the submitted version.

## Conflict of Interest

The authors declare that the research was conducted in the absence of any commercial or financial relationships that could be construed as a potential conflict of interest.
