# Supplementary material for: A Systematic Review and Meta-Analysis of Health Utility Estimates in Chronic Spontaneous Urticaria
Source: Front Med (Lausanne). 2020 Dec 9;7:543290. doi: 10.3389/fmed.2020.543290 (PMC7793814; doi:10.3389/fmed.2020.543290)
Supplement: Supplementary file 1 [file Data_Sheet_1.docx]

**Table S1. PRISMA CHECKLIST**

| **Section/topic** | **#** | **Checklist item** | **Reported on page #** |
| --- | --- | --- | --- |
| **TITLE** | | |  |
| Title | 1 | Identify the report as a systematic review, meta-analysis, or both. | Page 1 (Title): A systematic review and meta-analysis of utility-based quality of life in chronic spontaneous urticaria |
| **ABSTRACT** | | |  |
| Structured summary | 2 | Provide a structured summary including, as applicable: background; objectives; data sources; study eligibility criteria, participants, and interventions; study appraisal and synthesis methods; results; limitations; conclusions and implications of key findings; systematic review registration number. | Page 3 (Abstract) |
| **INTRODUCTION** | | |  |
| Rationale | 3 | Describe the rationale for the review in the context of what is already known. | Page 4 paragraph 1, 2 |
| Objectives | 4 | Provide an explicit statement of questions being addressed with reference to participants, interventions, comparisons, outcomes, and study design (PICOS). | Page 5, paragraph 3 |
| **METHODS** | | |  |
| Protocol and registration | 5 | Indicate if a review protocol exists, if and where it can be accessed (e.g., Web address), and, if available, provide registration information including registration number. | Page 5, paragraph 3*.* |
| Eligibility criteria | 6 | Specify study characteristics (e.g., PICOS, length of follow-up) and report characteristics (e.g., years considered, language, publication status) used as criteria for eligibility, giving rationale. | Page 5, paragraph 3*.* |
| Information sources | 7 | Describe all information sources (e.g., databases with dates of coverage, contact with study authors to identify additional studies) in the search and date last searched. | Page 5, paragraph 3*.* |
| Search | 8 | Present full electronic search strategy for at least one database, including any limits used, such that it could be repeated. | Table S2 |
| Study selection | 9 | State the process for selecting studies (i.e., screening, eligibility, included in systematic review, and, if applicable, included in the meta-analysis). | Page 5, paragraph 3*.* |
| Data collection process | 10 | Describe method of data extraction from reports (e.g., piloted forms, independently, in duplicate) and any processes for obtaining and confirming data from investigators. | Page 5, paragraph 3. |
| Data items | 11 | List and define all variables for which data were sought (e.g., PICOS, funding sources) and any assumptions and simplifications made. | Page 5, paragraph 3. |
| Risk of bias in individual studies | 12 | Describe methods used for assessing risk of bias of individual studies (including specification of whether this was done at the study or outcome level), and how this information is to be used in any data synthesis. | Page 6, paragraph 2. |
| Summary measures | 13 | State the principal summary measures (e.g., risk ratio, difference in means). | Page 6, paragraph 2. |
| Synthesis of results | 14 | Describe the methods of handling data and combining results of studies, if done, including measures of consistency (e.g., I^2^) for each meta-analysis. | Page 7, paragraph 4. |
| Risk of bias across studies | 15 | Specify any assessment of risk of bias that may affect the cumulative evidence (e.g., publication bias, selective reporting within studies). | Page 6, paragraph 2. |
| Additional analyses | 16 | Describe methods of additional analyses (e.g., sensitivity or subgroup analyses, meta-regression), if done, indicating which were pre-specified. | Page 6, paragraph 2. |
| **RESULTS** |  |  |  |
| Study selection | 17 | Give numbers of studies screened, assessed for eligibility, and included in the review, with reasons for exclusions at each stage, ideally with a flow diagram. | Figure 1 |
| Study characteristics | 18 | For each study, present characteristics for which data were extracted (e.g., study size, PICOS, follow-up period) and provide the citations. | Table 1, 2 |
| Risk of bias within studies | 19 | Present data on risk of bias of each study and, if available, any outcome level assessment (see item 12). | Figure S1 |
| Results of individual studies | 20 | For all outcomes considered (benefits or harms), present, for each study: (a) simple summary data for each intervention group (b) effect estimates and confidence intervals, ideally with a forest plot. | Page 6, paragraph 3. |
| Synthesis of results | 21 | Present results of each meta-analysis done, including confidence intervals and measures of consistency. | Table 1, 2, Figure 2,3 |
| Risk of bias across studies | 22 | Present results of any assessment of risk of bias across studies (see Item 15). | Figure S1 |
| Additional analysis | 23 | Give results of additional analyses, if done (e.g., sensitivity or subgroup analyses, meta-regression [see Item 16]). | Figure 1-Figure 5,Figure S1-S2 |
| **DISCUSSION** |  |  |  |
| Summary of evidence | 24 | Summarize the main findings including the strength of evidence for each main outcome; consider their relevance to key groups (e.g., healthcare providers, users, and policy makers). | Page 8 paragraph 3. |
| Limitations | 25 | Discuss limitations at study and outcome level (e.g., risk of bias), and at review-level (e.g., incomplete retrieval of identified research, reporting bias). | Page 8, paragraph 4 |
| Conclusions | 26 | Provide a general interpretation of the results in the context of other evidence, and implications for future research. | Page 10, paragraph 2 |
| **FUNDING** |  |  |  |
| Funding | 27 | Describe sources of funding for the systematic review and other support (e.g., supply of data); role of funders for the systematic review. | Page 2, paragraph 2 |

**Table S2.**

**Population search**

| **MESH/subject headings** | **Text words** |
| --- | --- |
| Urticaria | Hives |

**Utility search**

| **MESH/subject headings** | **Text words continued** |
| --- | --- |
| Quality adjusted life years | SF 36 |
|  | SF6D |
| **Text words** | SF 6D |
| AQOL | SF12 |
| Cost utility analysis | SF 12 |
| CUA | 15D |
| DALY | Standard gamble |
| Disability adjusted life year | Time trade off |
| EuroQol 5D | TTO |
| EQ5D | Utility analysis |
| EQ 5D | Utility assessment |
| Health utility index | Utility based quality of life |
| HUI | Utility estimat |
| QALY | Utility score |
| Quality Adjusted life year |  |
| Preference based quality of life |  |
| Preference elicitation |  |

**Table S3. Quality assessment of the included studies**

| Study | Selection | | | |  | | Comparability | |  | Exposure | | |  | Score |
| --- | --- | --- | --- | --- | --- | --- | --- | --- | --- | --- | --- | --- | --- | --- |
|  | 1 | 2 | 3 | 4 | |  | 5a | 5b |  | 6 | 7 | 8 |  |  |
| Baiardini et al.2003 | √ | √ | × | √ | |  | √ | √ |  | × | √ | √ |  | 7 |
| Ozkan et al.2007 | √ | √ | √ | √ | |  | √ | √ |  | × | √ | √ |  | 8 |
| Balp et al.2014 | √ | × | √ | √ | |  | √ | × |  | × | √ | √ |  | 6 |
| Balp et al.2015 | √ | × | √ | √ | |  | √ | √ |  | × | √ | √ |  | 7 |
| McBride et al.2015 | √ | × | × | × | |  | √ | × |  | × | √ | × |  | 3 |
| Sussman et al.2015 | √ | × | × | × | |  | √ | × |  | × | √ | × |  | 3 |
| Vietri et al.2015 | √ | √ | √ | √ | |  | √ | √ |  | × | √ | √ |  | 8 |
| Hawe et al.2016 | √ | √ | × | × | |  | √ | × |  | × | √ | √ |  | 5 |
| Balp et al.2017 | √ | √ | × | √ | |  | √ | √ |  | × | √ | √ |  | 7 |
| Mendelson et al.2017 | √ | × | × | √ | |  | √ | √ |  | × | √ | √ |  | 6 |
| Balp et al.2018 | √ | × | × | √ | |  | √ | √ |  | × | √ | √ |  | 6 |
| Nguyen et al.2019 | √ | √ | × | √ | |  | √ | √ |  | × | √ | √ |  | 7 |

1. Definition of case; 2. Representativeness of the cases; 3. Selection of controls; 4. Definition of controls; 5a. Study controls for utility elicitation instrument; 5b. Study controls for additional factor; 6. Ascertainment of exposure; 7. Same method of ascertainment for cases and controls; 8. Non-response rate.

Supplementary Figure 1.


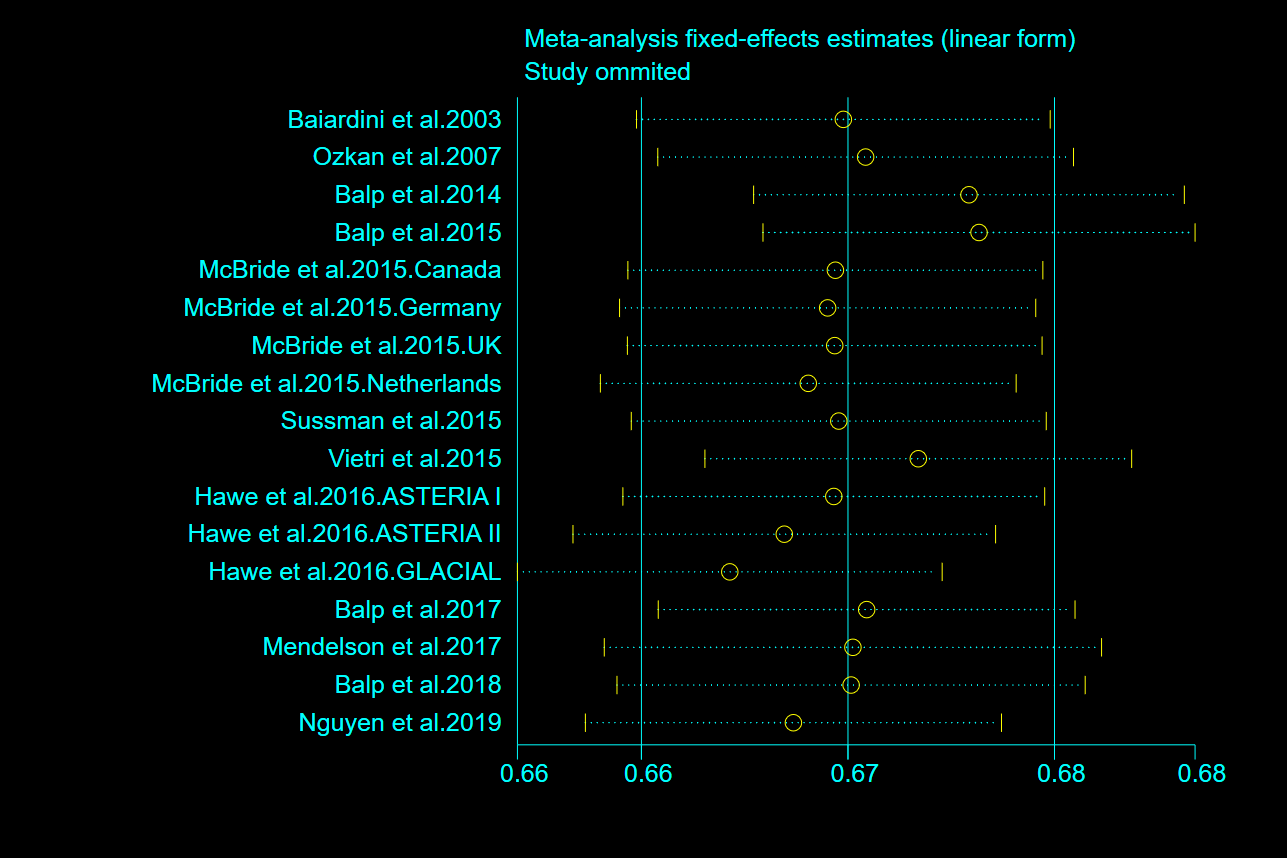


**Supplementary file captions**

Figure S1. Sensitivity analysis of studies.

Table S1. PRISMA CHECKLIST

Table S2. Summary of terms used in the Medline search strategy.

Table S3. Quality assessment of the included studies.
